# Bactericidal and Anti-biofilm Activity of the Retinoid Compound CD437 Against *Enterococcus faecalis*

**DOI:** 10.3389/fmicb.2019.02301

**Published:** 2019-10-09

**Authors:** Fang Tan, Pengfei She, Linying Zhou, Yiqing Liu, Lihua Chen, Zhen Luo, Yong Wu

**Affiliations:** Department of Medicine Clinical Laboratory, The Third Xiangya Hospital of Central South University, Changsha, China

**Keywords:** retinoid, *Enterococcus faecalis*, antibacterial, resistance, biofilm, synergism

## Abstract

*Enterococcus faecalis* (*E. faecalis*), a biofilm-forming pathogen, causes nosocomial infections. In recent years, drug resistance by enterococci has become increasingly severe due to widespread antibiotic abuse. Therefore, novel antibacterial agents are urgently needed. In this study, the synthetic retinoid compound CD437 was found to have potent bactericidal effect on *E. faecalis*. In addition, CD437 exhibited synergistic effects when administered in combination with gentamicin and additive effects when combined with ceftriaxone sodium. CD437 also inhibited biofilm formation by *E. faecalis* and exerted bactericidal effect on mature biofilm. Moreover, CD437 exhibited antibacterial and anti-biofilm effects against *Staphylococcus*. No bactericidal action of CD437 was observed against the gram-negative bacillus, but *Pseudomonas aeruginosa* biofilm extracellular polymeric substances (EPS) matrix formation was reduced. Overall, these findings indicate that CD437 has the potential to be developed as a novel antibacterial drug.

## Introduction

*Enterococcus faecalis* (*E. faecalis*) is a gram-positive, facultative anaerobic oval coccus that can form chains of varying length. It can survive under harsh conditions, including high salt concentrations and a range of temperatures (from 10°C to >45°C) (Arias and Murray, 2012). It is widely distributed in the nature and the gastrointestinal tract of humans, animals, and insects. *E. faecalis* is an important pathogen of nosocomial infections, mainly causing urinary tract infections, bacteremia, artificial joint infections, abdominal–pelvic infections, and endocarditis (Arias et al., 2010; Tornero et al., 2014).

Because antibacterial-drug use has increased rapidly in recent years, enterococci resistance has become increasingly serious and prevalent. Therefore, several drugs that target gram-positive bacteria are not effective against *E. faecalis*. Intrinsic properties of *Enterococcus* cause resistance against common antibiotics, such as the production of the low-affinity penicillin-binding protein Pbp5 that results in reduced sensitivity to penicillin and ampicillin (Kristich et al., 2014). Poor cell wall permeability makes *Enterococcus* highly resistant to clinically achievable aminoglycoside concentrations, rendering this antibiotic unusable as a single agent (Garcia-Solache and Rice, 2019). Enterococcal resistance to linezolid is associated with mutations in the central loop of domain V of the 23S rRNA (Mendes et al., 2014). In addition, the transferable oxazolidinone resistance gene *cfr* mediates acquired drug resistance (Diaz et al., 2012). Glycopeptide-resistant enterococci are known to synthesize new peptidoglycan precursors, including D-Ala-D-lactate or D-Ala-D-Ser, which replace the normal D-Ala-D-Ala termini (Depardieu et al., 2007). Nine gene clusters that cause glycopeptide resistance have been found in *Enterococcus*, including VanA- and VanB-type resistance clusters (Garcia-Solache and Rice, 2019).

Biofilms are communities of microorganisms encased in extracellular polymeric substances (EPS) (Karatan and Watnick, 2009). The formation of biofilms makes bacteria more capable of adapting to the external environment, which can increase resistance to antibiotics by 1000-fold (Coenye and Nelis, 2010; Pereira et al., 2014), and it is estimated that approximately 65–80% of infections in humans are biofilm mediated. Bacteria can adhere to the heart valves, wounds, and various catheters (Pereira et al., 2014). In *E. faecalis*, biofilm formation commonly occurs on urinary catheters, which can result in a strong immune response (Stickler, 2008; Guiton et al., 2010).

In addition to drug resistance and biofilm formation, the production of bacterial toxins and enzymes increases treatment difficulty for enterococci infections. *E. faecalis* expresses toxins such as lysin and hemolysin, which contribute to cell lysis and virulence, and produces gelatinase and serine protease that can degrade host tissues, regulate biofilm development, and promote microbial invasion. Additionally, adhesin and collagen-binding proteins of *E. faecalis* support biofilm formation in intravascular infections (Kristich et al., 2014). These factors complicate the treatment of *E. faecalis* infections and increase the urgency for new antibiotics.

Recently, it has been reported that the synthetic retinoid compound CD437 can effectively kill growing and persistent methicillin-resistant *Staphylococcus aureus* (MRSA) cells by disrupting the lipid bilayer (Kim et al., 2018). We also speculated that CD437 has bactericidal effect on *E. faecalis*, another gram-positive coccus. In this study, we investigated whether CD437 can inhibit the formation of bacterial biofilms and whether it can eradicate highly resistant biofilms. In addition, we examined the bactericidal action of CD437 and determined its effect in combination with antibiotics.

## Materials and Methods

### Bacterial Isolates, Cultural Conditions, and Reagents

*E. faecalis* ATCC 29212, *A. baumannii* ATCC 1195, *K. pneumoniae* ATCC 700603, *E. coli* ATCC 25922, *P. aeruginosa* PAO1 (ATCC 15692) were obtained from the American Type Culture Collection, *S. epidermidis* RP62A (ATCC 35984) and ATCC 12228 were given by Di Qu (Shanghai Medical College of Fudan University), and *S. aureus* RJ-2 (ST59, clinical isolate of MRSA) were provided by Li Min (Shanghai Jiao Tong University). The clinical isolates of *E. faecium*, *S. epidermidis*, and *P. aeruginosa* were collected from patients in the Third Xiangya Hospital of Central South University, China. *S. aureus*, *S. epidermidis*, and *E. faecium* strains were grown in tryptic soy broth (TSB) (Solarbio, Beijing, China) or brain-heart infusion (BHI) broth (Solarbio), respectively, at 37°C. Other strains were grown in Luria Bertani (LB) broth (Solarbio). Vancomycin, gentamicin, levofloxacin, and ceftriaxone sodium were purchased from Aladdin (Shanghai, China). CD437 was purchased from MedChem Express (Monmouth Junction, NJ, United States). All compounds were prepared as 10 mg/mL stock solution in DMSO or double-distilled H_2_O (DDH_2_O).

### Minimal Inhibitory Concentration and Minimal Bactericidal Concentration Determination Assay

The minimal inhibitory concentration (MIC) was determined using the standard micro-dilution method recommended by the Clinical and Laboratory Standards Institute (Clinical and Laboratory Standards Institute [CLSI], 2012). To determine the minimal bactericidal concentration (MBC), bacterial cultures from wells with antibiotic concentrations equal to or higher than the MIC were streaked on blood agar plates and incubated at 37°C for 48 h. The MBC was defined as the lowest concentration for which no visible bacterial colonies were observed on the plates after 24 h (Huang et al., 2012). The assay was conducted in triplicate.

### Screening of Drug Resistance in Serial Passage

Serial-passage experiments were performed in 96-well cell culture microtiter plates (Corning costar, Cambridge, MA, United States) as a series of MIC experiments using a wide range of CD437 concentrations. CD437 was serially diluted in Mueller Hinton (CaMH) broth, and 50 μL of overnight *E. faecalis* suspension (approximately 10^6^ CFU/mL) was added to wells containing 50 μL of the serially diluted compound. The initial concentration of CD437 was twice that of the MIC. After incubation at 37°C for 24 h, optical density (OD) was measured at 630 nm using a spectrophotometer (iMark^TM^ Microplate Absorbance Reader, BIO-RAD, Hercules, CA, United States). Bacterial growth was defined as OD_630__nm_ > 0.1, and 2 μL of culture with the highest drug concentration that allowed bacterial growth was diluted 1000-fold in CaMH and used as an inoculum for the next passage. The remaining culture was stored in 16% glycerol at −80°C (Friedman et al., 2006). Reduced sensitivity to CD437 was determined by re-measuring the MIC of CD437 against resistant mutants from each glycerol-frozen parent. The same protocol was used for ceftriaxone sodium and vancomycin as the controls.

### Time-Kill Assay

A single *E. faecalis* colony was inoculated into 25 mL of BHI medium and grown overnight at 37°C, with shaking at 180 rpm. Bacterial suspension (5 mL) was collected in centrifuge tubes and centrifuged at 6000 × *g* for 10 min at 4°C. The sediments were then collected and re-suspended in the same volume of CD437 diluted in normal saline at concentrations of 0, 2, 4, and 8 μg/mL. Samples were collected at 0, 2, 4, 8, and 24 h, serial diluted 10-fold with saline, and spread on drug-free plates (Cox et al., 2000), After incubating at 37°C for 24 h, colonies were counted, and the number of viable cells is reported as colony forming units per mL (CFU/mL). The experiments were conducted in triplicate.

### Bacterial Growth Curves

A single *E. faecalis* colony was inoculated into 25 mL of BHI medium and grown overnight at 37°C, with shaking at 180 rpm. Bacterial suspension (500 μL of MCF 0.5) was inoculated in to 9.5 mL of BHI medium with CD437 at a concentration of 0.5 × MIC, 1 × MIC, or 2 × MIC, or without CD437, to a final bacterial concentration of ∼10^6^ CFU/mL. The cultures were incubated at 37°C, with shaking at 180 rpm, and 200 μL of the suspension was sampled every 2 h to determine the OD at 630 nm. Cultures were continuously monitored for 24 h, and the growth curve was plotted using OD_630__nm_ average values over three time points as ordinate and culture time as abscissa. The experiments were conducted in triplicate.

### Determination of Minimum Biofilm Inhibitory Concentration

The Minimum Biofilm Inhibitory Concentration (MBIC) is the lowest concentration of an agent that inhibits visible biofilm formation of a microorganism (Xu et al., 2012). The MBIC is measured based on the reduction of 2,3-bis(2-methoxy-4-nitro-5-sulfophenyl)-2H-tetrazolium-5-carboxanilide (XTT) to a water-soluble orange compound by biofilm cells (Pierce et al., 2008). Overnight culture broth (4 μL) was added to 196 μL of BHI medium containing diluted CD437 in a 96-well plate. A control well with 196 μL of medium and 4 μL of overnight culture broth and a blank control with only 200 μL of BHI medium were used. After incubation of the plate at 37°C for 24 h, the medium was discarded and the plate was washed twice with saline to remove any planktonic cells. For XTT staining (Gomes et al., 2009), XTT was diluted with 1 × PBS (pH = 7.0) to a final concentration of 0.2 mg/mL and mixed with PMS (0.02 mg/mL). The mixture (200 μL) was then added to each well, and after incubation at 37°C for 3 h in dark, OD_490__nm_ of sample in each well was measured. The experiments were conducted in triplicate.

### Assessment of Biofilm Biomass

The drug dilution process and biofilm culture were as described above. The 96-well plate was washed once with saline to remove planktonic cells, and then 200 μL of 0.25% crystal violet (CV) solution was added. Staining was carried out at room temperature for 15 min and the plate was washed three times with saline to remove excess dye (Stepanovic et al., 2000). During this washing step, precautions were taken to avoid damage to the biofilm. The plate was dried at 50°C for 30 min, and the bound dye was dissolved in each well for 20 min by adding 95% ethanol. The absorbance of the sample was then measured at 570 nm. The experiments were conducted in triplicate.

### Assessment of Viable Cells in Biofilm by Colony Count

Mature biofilm was formed by adding 196 μL of BHI medium to a 96-well plate, before adding 4 μL of overnight bacterial culture, and incubated at 37°C for 24 h. After 24 h, the medium was discarded, the plate was washed with saline, and CD437 diluted in BHI medium was added to each well. The plate was incubated at 37°C for 24 h, washed again, and 200 μL of saline was added to each well. The biofilm was broken up through vigorous pipetting to ensure detachment from wells. The bacterial suspension was then transferred to a new 96-well plate and serial 10-fold dilutions were performed in saline. Five microliters of each dilution was plated on agar plates and the CFU was enumerated after 24 h of incubation at 37°C. The experiment was performed twice with three replicates.

### Checkboard Assay

The MIC of the antibacterial drug to be tested was first determined separately. According to the obtained MIC, the drug concentration (generally 6–8 dilutions) was determined, and the highest concentration of drug used was twice the MIC. The dilution of the two drugs was carried out in longitudinal and horizontal rows of the 96-well plate, to ensure that a mixture of different concentrations of the two drugs was obtained in each well. The bacterial inoculation was adjusted to 5 × 10^5^ CFU/mL and incubated at 35°C for 18–24 h (Sueke et al., 2010). The assay was performed in triplicate. The fractional inhibitory concentration index (FICI) was calculated using the following formula:

$$\text{FICI}=\frac{\text{MIC}\,\left(A\,\mathrm{combination}\right)}{\text{MIC}\,\left(A\,\mathrm{alone}\right)}+\frac{\text{MIC}\,\left(B\,\mathrm{combination}\right)}{\text{MIC}\,\left(B\,\mathrm{alone}\right)}$$

Judgment criteria: FICI ≤ 0.5 is synergistic; 0.5 < FICI ≤ 1 is additive; 1 < FICI ≤ 4 is irrelevant; and >4 is antagonistic (Almaaytah et al., 2018).

### Assessment of CD437 Effect on Biofilm Morphology

Forty microliters of overnight culture was added to a 6-well cell culture plate (Corning costar, Cambridge, MA, United Status) containing 1960 μL of CD437 dissolved in BHI medium and a 18 mm × 18 mm sterile glass cover slide. After 24 h of incubation at 37°C without shaking, the slide was washed once with saline to wash away planktonic cells. Finally, slides were stained with the pre-mixed dyes SYTO9 (green) and PI (red; LIVE/DEAD BacLight Bacterial Viability Kit [L7012], Thermo Fisher Scientific, MA, United States), and then images were captured using a confocal laser scanning microscope (CLSM) (ZEISS LSM800, Jena, Germany). For mature biofilm eradication experiments, 24-h biofilms were treated with or without CD437 for another 24 h. When cover slides were stained with 0.25% CV, biofilms were visualized and photographed using a light microscope (Olympus CX31, Tokyo, Japan).

### Human Blood Hemolysis Assay

Blood of a clinically normal person was collected from the Department of Clinical Laboratory of the Third Xiangya Hospital, and then 2 mL of blood sample was centrifuged at 500 × *g* for 5 min. The supernatant was aspirated and replaced with 50 mL of PBS buffer (pH 7.4) and centrifuged as previously described. This step was performed twice to wash erythrocytes. Erythrocytes of 2–3 individuals were mixed and diluted to 4% with PBS, and then 100 μL of the suspension was added to 100 μL of CD437 of different concentrations prepared in PBS, 0.2% DMSO (negative control), or 2% Triton X-100 (positive control) in sterile EP (Eppendorf) tubes. The tubes were incubated at 37°C for 1 h, centrifuged at 500 × *g* for 5 min, and 100 μL of the supernatant was added to a 96-well plate, before measuring the absorbance at 450 nm. The hemolysis rate was calculated using the following formula:

$$\text{henolysis}\left(\%\right)=100\frac{{\text{A}}_{\text{sample}}-{\text{A}}_{0.1\%\,\mathrm{DMSO}}}{{\text{A}}_{\text{TritonX}-100}-{\text{A}}_{0.1\%\,\mathrm{DMSO}}}$$

The concentration of CD437 at which 50% hemolysis (HC_50_) was determined using GraphPad Prism 8 (GraphPad Software Inc., CA, United States). The assay was conducted in triplicate.

### Mammalian Cell Culture and Cytotoxicity Test

Human liver cancer cell lines HepG2, Bel-7404, and human umbilical vein endothelial cells (HUVECs) (ATCC, Manassas, VA, United States) were grown in DMEM medium supplemented with 10% FBS and 1% penicillin/streptomycin antibiotic. Human renal proximal tubular epithelium HK-2 and human colorectal cancer cell line HT-29 (ATCC, Manassas, VA, United States) were grown in DMEM/F12 medium supplemented with 10% FBS and 1% penicillin/streptomycin antibiotic. All the cells were maintained at 37°C with 5% CO_2_ under a humidified atmosphere. For the cytotoxicity assay, cell viability was tested using the Cell Counting Kit-8 (CCK-8, DojinDo, Japan). The cells were seeded in 96-well plates with 5,000 cells (100 μL) per well, allowed to attach for 6–8 h, and then exposed to CD437 at different concentrations (100 μL), prepared in PBS or 0.1% DMSO (negative control), for 24 h. CCK-8 (10 μL) was then added to each well and the plates were incubated at 37°C for 1–4 h. The absorbance was measured at 450 nm, and the cytotoxicity was calculated using the following formula:

$$\text{cytotoxicity}\left(\%\right)=100\left(1-\frac{{\text{A}}_{\text{sample}}-{\text{A}}_{\text{blank}}}{{\text{A}}_{0.1\%\,\mathrm{DMSO}}-{\text{A}}_{\text{blank}}}\right)$$

The 50% inhibitory concentration (IC_50_) value was determined using GraphPad Prism 8 (GraphPad Software Inc., CA, United States). The assay was carried out in triplicate.

### qPCR

Briefly, PAO1 were cultured in a 6-well plate and treated with or without CD437 for 24 h, and RNA was isolated using E.Z.N.A. Total RNA Kit II (Omega Bio-tek, Norcross, GA, United States), cDNA was prepared using TransScript All-in-One First-Strand cDNA Synthesis SuperMix for qPCR (Transgene, Beijing, China), qPCR was performed using TransStart Tip Green qPCR SuperMix (Transgene, Beijing, China), primers were used as follows: *PslA*-forward, CGCTCACGGTGATTATGTTC; *PslA*-reverse, TACATGAACAACAGCAGGCA; *PelA*-forward, ACAGCCAGGTAATGGACCTC; and *PelA*-reverse, AAGCTGTCCAGGGTATCGAG (Kim et al., 2015). All qPCR reactions were run in triplicate using a CFX96 Real-Time PCR Detection System (Bio-Rad Laboratories Ltd., Hemel Hempstead, United Kingdom), the following conditions were utilized: 94°C for 5 s, 40 cycles of 94°C for 5 s, 58°C for 15 s, and 72°C for 10 s, Fold change of each gene was normalized to that of the 16S RNA of *P. aeruginosa*. The untreated samples were used for calibration.

### Mouse Peritonitis Model

Specific pathogen-free, 6-week old female ICR mice (SJA Laboratory Animal Co., Ltd., Hunan, China) with a mean weight of 25 g were used in this study. Sterile rat fecal extract (SRFE) was prepared using crushed, dried rat feces by mixing with two volumes of normal saline and autoclaving at 121°C and 15 lb of pressure for 15 min. Then the mixture was centrifuged at 3500 × *g* at a temperature of 4°C, and the supernatant (100% SRFE) was filtered by 0.22 μm Millipore Express^®^ PES Membrane Filters. Bacteria for inoculation were grown overnight to stationary phase. The resulting cells were then pelleted at 3500 × *g* for 10 min at 4°C and resuspended in normal saline, and pelleted again as described above. The washed bacterial was resuspended in 12.5% SRFE (Pai et al., 2003) to obtain the final bacterial concentration of 1 × 10^8^ CFU/ml, and then injected into the mouse abdominal cavity in a final volume of 1 mL. CD437 was dissolved in a 1:1 solution of Kolliphor EL (BASF, Ludwigshafen, Germany) and ethanol and then diluted 1:10 in PBS to a final concentration of 30 mg/kg. At 24 h post-infection, groups of mice (*n* = 6) were treated with 30 mg/kg gentamicin s.c., 30 mg/kg CD437 i.p., or a combination of 30 mg/kg CD437 i.p. and 30 mg/kg gentamicin s.c. every 12 h for 3 days. Control mouse were injected with 200 μL of 10% Kolliphor EL/ethanol in PBS i.p. every 12 h for 3 days. After euthanizing mice, liver and spleen tissue was homogenized in PBS, The numbers of bacterial in the homogenates were counted by 10-fold serial dilution and spotting on blood agar plates. The bacterial load was counted as CFU/g tissue. This study was carried out in accordance with the Animal Welfare Act and National Institutes of Health guidelines for animal care and use, and all experimental protocols were approved by the IRB of Third Xiangya Hospital, Central South University.

### Statistical Analysis

The quantitative data are presented as mean ± standard deviation. A student’s t-test was used for between-group comparisons, and multi-group comparisons were performed using a one-way ANOVA. The statistical analyses were performed using SPSS 21.0 (SPSS Inc., Chicago, IL, United States). The results with *P*-values of < 0.05 were considered significant.

## Results

### Bactericidal Effect of CD437 on *E. faecalis* ATCC 29212

**FIGURE 1:**
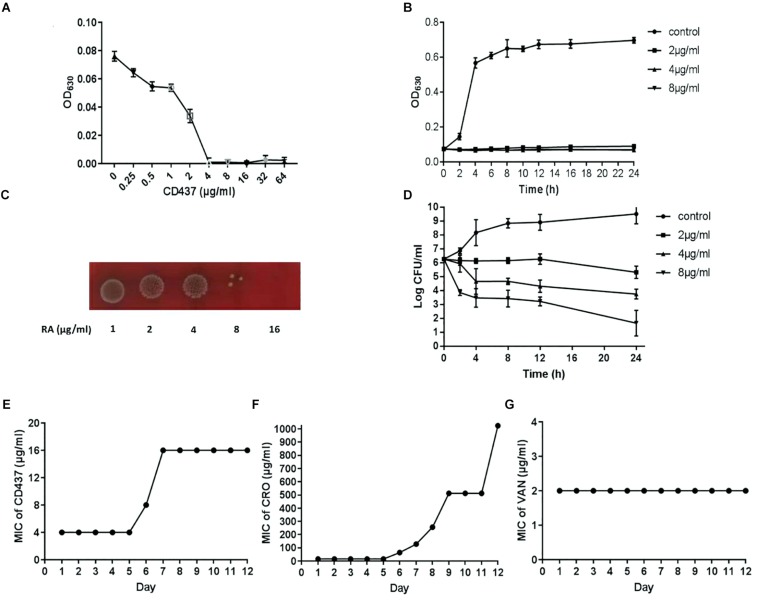
CD437 exerts a potent bactericidal effect on *E. faecalis* ATCC 29212. **(A)** Growth of *E. faecalis* ATCC 29212 after exposure to CD437 in BHI medium for 18 h. OD_630__nm_, optical density at 630 nm. **(B)** The growth curve shows that 2 μg/mL CD437 can significantly inhibit bacterial growth. **(C)** Bacterial cultures from the MIC test were streaked on agar and incubated for 48 h. **(D)** The time-kill curve shows that CD437 has a bactericidal mechanism of action, as evident by the drop in viable cell counts after 4 h. Data are presented as mean ± s.d. The results are representative of three independent experiments. Serial-passage experiments with *E. faecalis* ATCC 29212 selected for increasing resistance to **(E)** CD437, **(F)** ceftriaxone sodium, and **(G)** vancomycin.

The growth curve of *E. faecalis* ATCC 29212 showed that CD437 can effectively inhibit the growth of bacteria. CD437 partially inhibited bacterial growth at a concentration of 0.25 μg/mL, and the inhibitory effect increased with CD437 concentration. The MIC of CD437 against *E. faecalis* ATCC 29212 was 4 μg/mL (Figure 1A) and the MBC was 16 μg/mL (Figure 1C). The OD_630__nm_ value was not detectable until 16 h when the concentration of CD437 was 2 μg/mL, and at concentrations greater than 4 μg/mL, the bacteria did not grow at all (Figure 1B). The number of viable bacteria decreased from 10^6^ CFU/mL to 10^5^ CFU/mL after 4 h of treatment with 4 μg/mL CD437. Additionally, at a concentration of 8 μg/mL, the number of viable bacteria decreased rapidly to 10^5^ CFU/mL after 2 h and gradually to ∼10 CFU/mL after 24 h (Figure 1D). The results demonstrated that CD437 exerts bactericidal action in a relatively short time. Next, continuous culture under sub-inhibitory drug concentrations was employed to screen for resistant bacteria. During a passage over 12 consecutive days, the MIC of CD437 for *E. faecalis* ATCC 29212 increased from 4 to 16 μg/mL (Figure 1E), whereas that of ceftriaxone sodium increased from 16 to 1024 μg/mL, a 64-fold increase over 12 consecutive days (Figure 1F). Under the same culture conditions, the MIC of vancomycin did not change (Figure 1G). These results indicate that CD437 has good bactericidal effect, and the probability of inducing drug-resistant mutations is low.

### Effect of CD437 on Biofilm Formation of *E. faecalis* ATCC 29212

**FIGURE 2:**
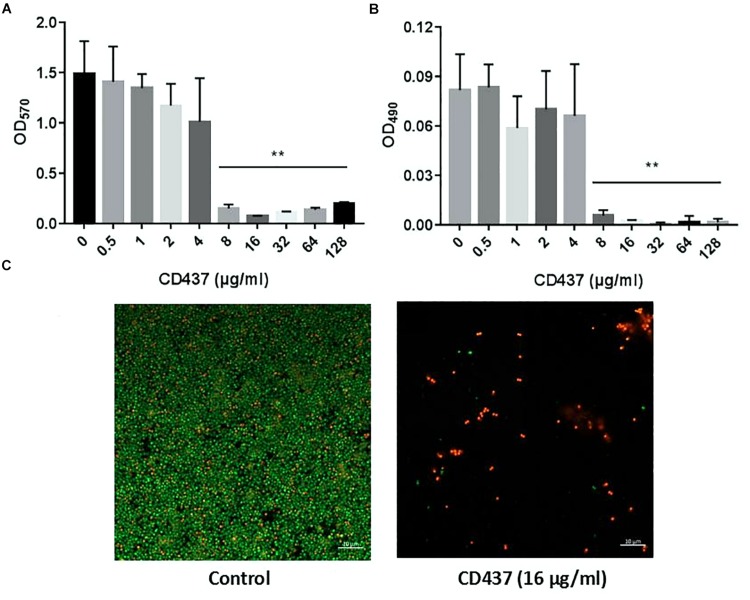
CD437 inhibits the formation of *E. faecalis* ATCC 29212 biofilm. **(A)** Different concentrations of CD437 were co-cultured with bacteria in 96-well plates at 37°C for 24 h. Biofilms were stained with crystal violet and quantified using the absorbance measured at 570 nm. **(B)** Biofilms were treated using the XTT assay and the absorbance was measured at 490 nm_._**(C)** The effect of CD437 on *E. faecalis* ATCC 29212 biofilm formation, according to the CLSM. Biofilms were formed on glass slides and stained with SYTO9 (green, live bacteria) and PI (red, dead bacteria). Data are presented as mean ± s.d. The results are representative of three independent experiments. ^∗∗^*P* < 0.01.

**FIGURE 3:**
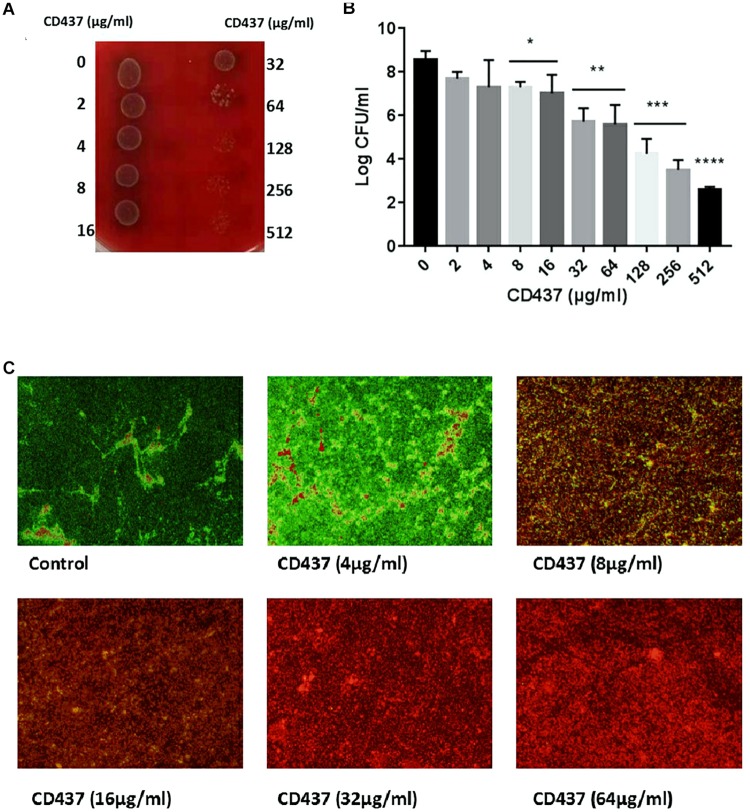
CD437 kills *E. faecalis* ATCC 29212 in mature biofilm. Mature biofilms (24-h-old) were treated with CD437 for another 24 h. **(A)** Biofilms were broken up by vigorous pipetting and detached from wells, and then 5 μL of bacterial suspension from each well was dropped onto blood agar before incubation at 37°C. **(B)** Live cells in biofilm were enumerated by colony count. **(C)** CD437 killed bacteria in mature biofilm on slides. Representative CLSM images of mature biofilms of *E. faecalis* ATCC 29212 on glass slides treated with CD437 for 24 h. Biofilms were stained with SYTO9 (green, live bacteria) and PI (red, dead bacteria). Images were taken at a magnification of 200×. Data are presented as mean ± s.d. (*n* = 3). ^∗^*P* < 0.05; ^∗∗^*P* < 0.01; ^∗∗∗^*P* < 0.001; and ^****^P < 0.0001.

Drug resistance can increase significantly with the formation of bacterial biofilm. CV staining can stain not only bacteria, but also biofilm extracellular matrix (including extracellular polysaccharide, protein, and DNA). CV staining showed that with increase in CD437 concentration, the biomass of biofilm decreased gradually, and when the concentration of CD437 was 8 μg/mL, biofilm formation was completely inhibited (Figure 2A). This result was consistent with the results of our XTT assay for detecting the metabolic activity of cells in biofilms (Figure 2B), which indicated that the MBIC was 8 μg/mL. The CLSM results showed that when the concentration of CD437 was two times the MBIC (16 μg/mL), very few bacteria adhered to the slide to form biofilm, and bacteria that did adhere were determined to be dead through staining with PI (red stain) (Figure 2C). CV staining in 96-well plates showed that CD437 could not eradicate mature biofilm, as the biomass did not decrease after treatment (Supplementary Figure S1). The number of viable cells in biofilm was also decreased in a dose-dependent manner (Figure 3A), and the colony count verified this result (Figure 3B). The CLSM showed that CD437 killed some cells in the mature biofilm at a concentration of 4 μg/mL, and as the concentration of CD437 increased, a greater number of red (dead) cells were observed. At a concentration of 64 μg/mL, almost all the biofilm cells were dead, and only a small number of green (live) cells were seen (Figure 3C). These results indicate that CD437 is capable of killing bacteria in biofilms, although it cannot eradicate them.

### Effect of CD437 and Antibiotic Combination on *E. faecalis* ATCC 29212

**FIGURE 4:**
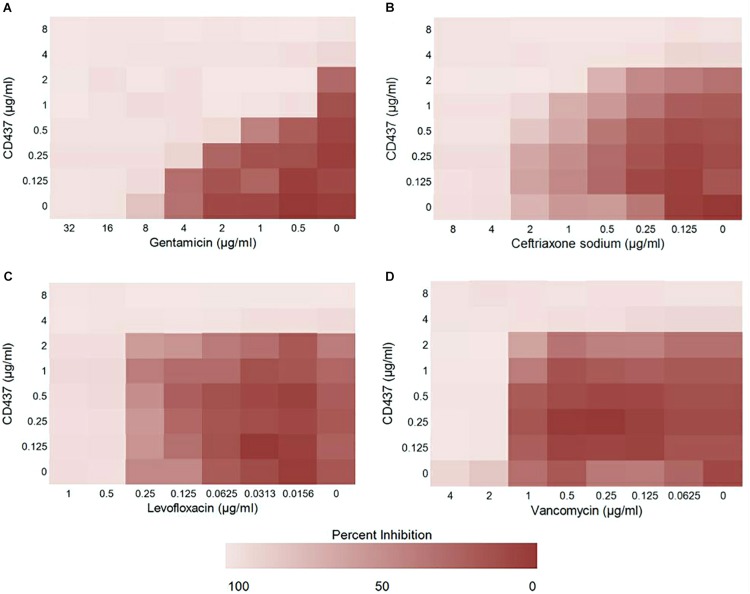
Results from the checkerboard assay. CD437 has a synergistic effect with **(A)** gentamicin and **(B)** ceftriaxone sodium against *E. faecalis* ATCC 29212. A synergistic effect was not observed with **(C)** levofloxacin or **(D)** vancomycin. Each assay was repeated three times, and the data are shown as the average change in OD_630__nm_.

To study the effect of CD437 on enterococci infection in combination with several antibiotics, we designed a checkerboard drug combination assay. The results showed that, when CD437 at a concentration of 1 μg/mL was combined with gentamicin, the MIC of gentamicin decreased from 8 to 0.25 μg/mL, a 32-fold decrease (Figure 4A). When CD437 was combined with ceftriaxone sodium, the addition of 2 μg/mL CD437 reduced the MIC of ceftriaxone sodium from 4 to 1 μg/mL, a 4-fold decrease (Figure 4B). However, when CD437 was combined with levofloxacin or vancomycin, the MIC of CD437 or the antibiotics did not decrease (Figures 4C,D). When the FICI was calculated, CD437 was found to have a synergistic effect when combined with gentamicin, with an FICI of 0.281. Additionally, CD437 had an additive effect when combined with ceftriaxone sodium, and the FICI was 0.75. The FICI was greater than 1 when CD437 was combined with levofloxacin and vancomycin, indicating that CD437 is ineffective in the presence of ofloxacin and vancomycin (Table 1).

**TABLE 1:** Combined effect of CD437 with antibiotics.

**Antibacterial agent**	**Class**	**MIC_A_ (ug/mL)**		**MIC_B_ (ug/mL)**		**FICI**	**Outcome**
		**Alone**	**Combination**	**Alone**	**Combination**		
Gentamicin	Aminoglycoside	8	0.25	4	1	0.281	Synergy
Ceftriaxone sodium	Cephalosporin	16	4	4	2	0.75	Additivity
Levofloxacin	Quinolones	0.5	0.5	4	2	1.5	No interaction
Vancomycin	Glycopeptide	2	1	4	4	1.5	No interaction

*MIC_*A*_ is the minimum inhibitory concentration of the antibiotic; MIC_*B*_ is the minimum inhibitory concentration of CD437; and FICI is the fractional inhibitory concentration index.*

### Effect of CD437 on Gram-Positive Cocci and Gram-Negative Bacilli

**FIGURE 5:**
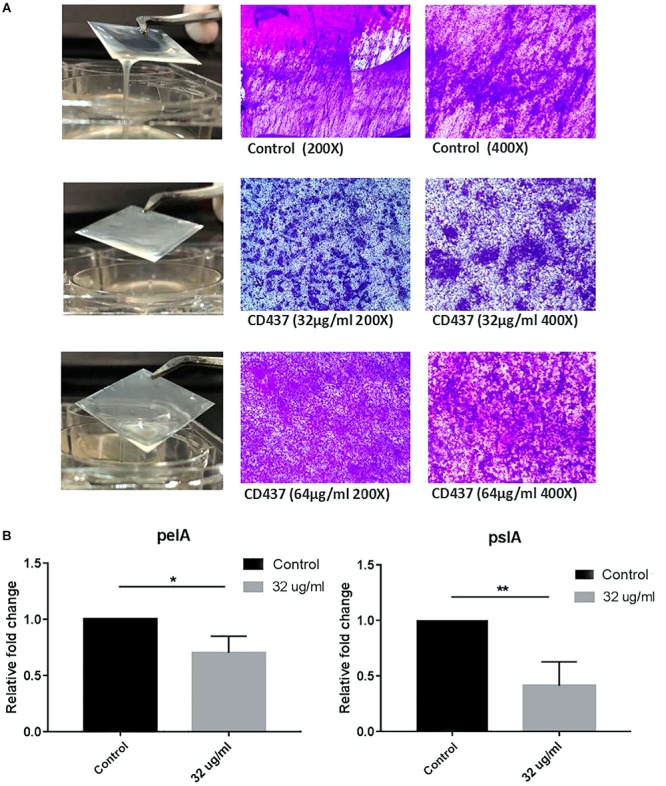
CD437 reduces the formation of PAO1 biofilm matrix, but does not inhibit bacterial adhesion. **(A)** Representative images of 0, 16, and 32 μg/mL of CD437 applied to the PAO1 biofilm. Biofilms were stained with CV. **(B)** Relative expression of *pelA* and *pslA*. PAO1 were cultured with or without CD437 for 24 h, and the transcription of *pelA*, *pslA* was measured by qPCR. ^∗^*P* < 0.05; ^∗∗^*P* < 0.01.

The MIC of CD437 for four *E. faecalis* clinical strains was 4 μg/mL, while the MBIC was 8 μg/mL. The MIC for *S. aureus* RJ-2 was 2 μg/mL and the MBIC was 4 μg/mL. The MIC and MBIC for biofilm-positive *S. epidermidis* RP62A were both 4 μg/mL, and the MIC for biofilm-negative *S. epidermidis* ATCC 12228 was 2 μg/mL, indicating that CD437 had a potent bacteriostatic effect on *Staphylococcus*. CD437 had no effect on *P. aeruginosa* PAO1 (Table 2). The MBIC test showed that CD437 effectively inhibited biofilm formation by *S. epidermidis*, *S. aureus*, and *E. faecalis*, but exerted no effect on gram-negative bacilli (Table 2). However, when PAO1 biofilm present on a glass slide in a 6-well plate was incubated with CD437, biofilm matrix was found to be reduced. The control biofilm produced a viscous extracellular matrix, and when the slides were picked up with a pair of tweezers, wire drawings appeared, but this phenomenon was not observed with PAO1 biofilm treated with CD437. When the slides were then subjected to CV staining, the biofilms of the control group were reticulated and cross-linked, while the CD437-treated biofilm bacteria formed mushroom-like aggregates (Figure 5), and this was more evident in the CLSM observation of PAO1 (Supplementary Figure S2). In the clinical isolate of *P. aeruginosa*, the above similar phenomena were noticed but their biofilm formation ability was lower than PAO1 and the matrix reduction was not clear as that of PAO1 (Supplementary Figure S3). However, during semi-quantitative CV staining of 96-well plates, there was no significant change in OD_570__nm_ (Supplementary Figure S4). Therefore, CD437 did not inhibit the growth and adhesion of *P. aeruginosa*, nor did it reduce the total biomass of *P. aeruginosa* biofilm. There is a research which suggested that PAO1 biofilm matrix thickness was associated with exopolysaccharide, not extracellular DNA, in particular, Pel and Psl (Lee et al., 2017). Pel and Psl polysaccharide production was indirectly measured by qPCR analysis of *pslA* and *pelA*, which are the constituents of the psl and pel operons, respectively. The results showed that the relative expression levels of *pelA* and *pslA* genes of CD437-treated PAO1 biofilm bacteria decreased as compared with the control group (*P* < 0.05) (Figure 5B). When CD437 is combined with gentamicin, ceftriaxone sodium, levofloxacin, and ciprofloxacin, it could not reduce the MBIC of these antibiotics to PAO1(Supplementary Table S1).

**TABLE 2:** MIC and MBIC of CD437 for gram-positive cocci and gram-negative bacilli.

**Strain**	**Type**	**MIC (ug/mL)**	**MBIC (ug/mL)**
*S. epidermidis* RP62A	ATCC 35984	4	4
*S. epidermidis* 12228 (BF^–^)	ATCC 12228	2	/
*S. epidermidis* 1	Clinical isolate	4	4
*S. epidermidis* 5	Clinical isolate	2	4
*S. epidermidis* 10 (BF^–^)	Clinical isolate	4	/
*S. epidermidis* 11 (BF^–^)	Clinical isolate	4	/
*S. aureus* RJ-2	Clinical isolate (MRSA)	2	4
*E. faecalis* 1	Clinical isolate	4	8
*E. faecalis* 9	Clinical isolate	4	8
*E. faecalis* 11	Clinical isolate	4	8
*E. faecalis* 13	Clinical isolate	4	8
*A. baumannii*	ATCC 1195	>64	>64
*K. pneumoniae*	ATCC 700603	>64	>64
*E. coli*	ATCC 25922	>64	>64
*P. aeruginosa* PAO1	ATCC 15692	>64	>64
*P. aeruginosa* 7	Clinical isolate	>64	>64
*P. aeruginosa* 14	Clinical isolate	>64	>64
*P. Aeruginosa* 17	Clinical isolate	>64	>64
*P. Aeruginosa* 47	Clinical isolate	>64	>64

*BF^–^ is negative for biofilm formation.*

### Mammalian Cytotoxicity and Hemolytic Activity

**FIGURE 6:**
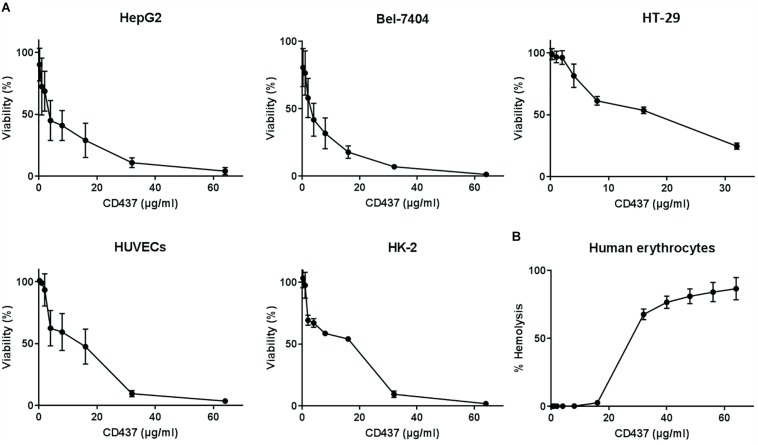
Mammalian cytotoxicity and hemolytic activity. **(A)** Human liver cancer cell lines HepG2 and Bel-7404, human colorectal cancer cell line HT-29, human umbilical vein endothelial cells (HUVECs), and human renal proximal tubular epithelium cell line HK-2 were treated with two-fold serially diluted concentrations of CD437 for 24 h. CCK-8 was added and incubated for 3h, and the absorbance at OD_450__nm_ was measured to calculate cellular activity. **(B)** 2% human erythrocytes were treated with CD437 and 1% Triton X-100 was used as a positive control. The absorbance of the supernatants was measured at 540 nm.

The HC_50_ of CD437 for human erythrocytes was 25.95 μg/mL, while the IC_50_ value of CD437 for human liver cancer cell lines HepG2 and Bel-7404 was 3.834 and 4.951 μg/mL, respectively. The IC_50_ value for human colorectal cancer cell line HT-29 was 10.62 μg/mL. The IC_50_ value of CD437 for HUVECs and human renal proximal tubular epithelium HK-2 was 16.48 and 19.52 μg/mL, respectively (Figure 6 and Table 3). These results show that CD437 is more toxic against human cancer cells than non-cancerous cells. The IC_50_ value of CD437 against human non-cancerous cells was greater than the MIC (4 μg/mL) and MBIC (8 μg/mL) of CD437 against *E. faecalis*.

**TABLE 3:** IC_50_ values of CD437 for human cell lines.

**Cell lines**	**Human cancer cell lines**			**Human normal cell line**	
	**HepG2**	**Bel-7404**	**HT-29**	**HK-2**	**HUVECs**
IC_50_ (ug/mL)	3.834	4.951	10.62	19.52	16.48

*HepG2, human liver cancer cell line; Bel-7404, human liver cancer cell line; HT-29, human colorectal cancer cell line; HUVEC, human umbilical vein endothelial cell; HK-2, human renal proximal tubular epithelium. The results represent the average of triplicates.*

### Evaluation of the Antibacterial Activities in a Mice Peritonitis Model

**FIGURE 7:**
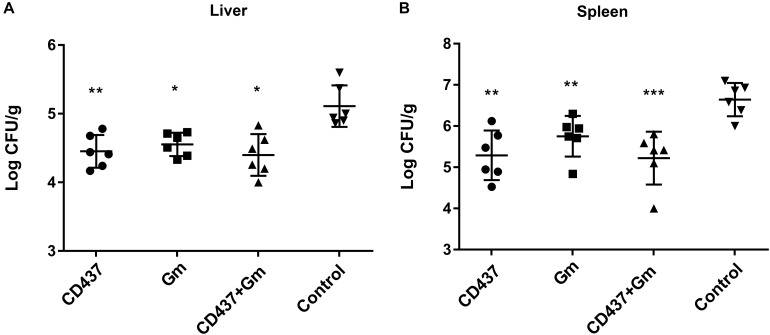
Efficacy of CD437 or in combination with gentamicin in mouse peritonitis model. 24 h after infection, Each group of *E. faecali*s-infected mice (*n* = 6) was treated with 30 mg/kg doses of CD437 (i.p.) alone or in combination with 30 mg/kg (s.c.) gentamicin (Gm), or control (5% Kolliphor + 5% ethanol, i.p.) every 12 h for 3 days. At 12 h after the last treatment, mice were euthanized and their **(A)** liver and **(B)** spleen were excised and homogenized. CFUs from each mouse are plotted as individual points and counted. The mean ± s.d. is displayed. ^∗^*P* < 0.05; ^∗∗^*P* < 0.01; and ^∗∗∗^*P* < 0.001.

The *in vivo* efficacy of CD437 were evaluated in a mouse peritonitis model. Six-week-old ICR female mice were intraperitoneally injected with 1 × 10^8^ colony-forming units (CFUs) of *E. faecalis* ATCC 29212 in 12.5% SRFE. As shown in Figure 7, compared to control group, *E. faecalis* abundance decreased approximately 5-fold in liver (*P* < 0.01) by 30 mg/kg CD437 alone while approximately 14-fold decrease in spleen (*P* < 0.01). On the other hand, 30 mg/kg gentamicin alone led to 4-fold decrease in bacterial load in liver (*P* < 0.05), and about 7-fold decrease in spleen (*P* < 0.01). 30 mg/kg CD437 in combination with 30 mg/kg gentamicin resulted in approximately 5-fold decrease in bacterial load in liver (*P* < 0.01), and around 21-fold decrease in spleen (*P* < 0.001). These results suggested that CD437 is effective *in vivo*.

## Discussion

*Enterococcus* can survive for long periods on environmental surfaces, such as medical equipment, bed rails, and door handles. These bacteria are resistant to heat, chlorine, and some alcoholic agents; additionally, *Enterococcus* often forms biofilm, making treatment difficult.

CD437 is a retinoid-like small molecule originally identified as a selective retinoic acid receptor γ (RARγ) agonist (Han et al., 2016). It induces cancer cell apoptosis through an unknown mechanism, while exerting no effect on non-cancerous cells. CD437 is toxic to several cancer cell lines derived from primary tumors, including ovarian cancer, non-small cell lung cancer, leukemia, breast cancer, and squamous cell carcinoma (Schadendorf et al., 1996; Sun et al., 1997; Hsu et al., 1999; Holmes et al., 2003; Rees et al., 2016). The most appealing feature of the use of CD437 as an anti-cancer drug is its selective toxicity against cancer cells and its low toxicity against normal cells (Han et al., 2016). Our data confirmed these results of previous studies. Additionally, A recent study has shown that CD437 can inhibit hepatitis B virus DNA amplification. CD437 directly inhibits DNA polymerase α (Pol α) (Han et al., 2016), and Pol α is vital for intracellular amplification of covalently closed circular DNA (cccDNA) (Tang et al., 2019). These findings indicate that CD437 could have broader therapeutic applications, extending beyond killing cancer cells.

The MIC of CD437 for *E. faecalis* ATCC 29212 and clinical strains of *E. faecalis* was 4 μg/mL, and the MBIC was 8 μg/mL, which was lower than the IC_50_ and HC_50_ values of CD437. These results suggest that CD437 could be a strong candidate for a new bactericidal drug. Although CD437 cannot eradicate an already formed mature biofilm, it can partially kill some bacteria in the biofilm at a relative low concentration. *E. faecalis* is inherently resistant to several antibiotics and readily accumulates mutations, which further contributes to resistance. In recent years, vancomycin-resistant enterococci have emerged, causing difficulty in treatment. Continuous culture of *E. faecalis* under sub-inhibitory drug concentrations of CD437 showed that CD437 had a relatively low incidence of drug-resistant mutations, compared with the mutation rate when cultured with ceftriaxone sodium.

Combination therapy is often required to treat complex enterococcal infections, in addition to infections of high inoculum and those with biofilm. For non-complex infections, monotherapy is generally sufficient. Endocarditis and meningitis infections caused by *Enterococcus* are usually treated with penicillin or ampicillin in combination with aminoglycoside antibiotics (Mercuro et al., 2018). In this study, the checkerboard assay showed that CD437 had a significant synergistic effect when combined with the aminoglycoside antibiotic gentamicin and had an additive effect when combined with the β-lactam antibiotic ceftriaxone sodium. CD437 alone or in combination with gentamicin also exhibited efficacy in a mice peritonitis model. Gram-positive bacteria contain a single inner phospholipid bilayer, while gram-negative bacteria have two lipid bilayers, and these membranes consist of three primary families of phospholipids: phosphatidylethanolamine (70–80% of total lipids), phosphatidylglycerol (20–25% of total lipids), and cardiolipin (5–10% of total lipids) (Auer and Weibel, 2017). CD437 has been reported to kill MRSA cells by destroying the cell membrane lipid bilayer (Kim et al., 2018). As evident above, we speculate that CD437 may kill *E. faecalis* using the same mechanism. Aminoglycoside antibiotics mainly act on the 30S subunit of the bacterial ribosome, which affects bacterial protein synthesis. Therefore, the synergistic effect of CD437 and gentamicin is likely to be the result of increased gentamicin diffusion through the bacterial cell membrane that has been physically damaged by CD437.

CD437 exhibited no bactericidal effect on gram-negative bacilli, including *K. pneumoniae*, *A. baumannii*, *E.coli*, and *P. aeruginosa* (Table 2). In this study, we confirmed that CD437 has no inhibitory effect on the biomass (CV staining) of *P. aeruginosa* biofilm. One explanation for this result could be that, in the 96-well plate semi-quantitative CV staining process, the viscous matrix is easily removed during the washing process. This effect may result in no detectable change in OD_570__nm_ between wells. However, in the slide-established biofilm, we found that CD437 inhibited extracellular polymeric substance matrix formation of PAO1, although it did not have an effect on bacterial adhesion and growth. The reduction in matrix is due to the low expression of *pelA* and *pslA*, which are the components of the operons for the synthesis of pel and psl polysaccharides.

In summary, CD437 has potent bactericidal effect on *E. faecalis*, and it not only inhibits the formation of *E. faecalis* biofilm but also has a killing effect on mature biofilms that are already formed. Additionally, CD437 has bactericidal action against *Staphylococcus* and inhibits the formation of biofilm. While CD437 exhibits no bactericidal effect on *P. aeruginosa*, it reduces the production of its extracellular polymeric substance matrix. Our study suggests that the retinoid compound CD437 has the potential to be further developed as a novel antibacterial drug.

## Data Availability Statement

The raw data supporting the conclusion of this manuscript will be made available by the authors, without undue reservation, to any qualified researcher.

## Ethics Statement

This study was carried out in accordance with the Animal Welfare Act and the National Institutes of Health guidelines for animal care and use, and all experimental protocols were approved by the IRB of the Third Xiangya Hospital, Central South University (No: 2015-S023). Strains and blood were isolated from clinical samples routinely collected from patients, and the identification of patients was not needed. Therefore, the need for written informed consent was waived and oral informed consent was obtained.

## Standard Biosecurity and Institutional Safety Procedures

All the biosafety measurements have been adopted and the institutional safety procedures are adhered. The laboratory of our institution has biosafety level 2 (BSL-2) standard where all standards and protocols are adopted as per the guidelines of CLSI.

## Author Contributions

YW, PS, and FT designed the experiments. FT performed most of the experiments, analyzed the results, and wrote the manuscript. LC and ZL provided the essential reagents and methods. LZ and YL performed the supporting experiments. YW conceived and supervised the study. All the authors read and approved the final manuscript.

## Conflict of Interest

The authors declare that the research was conducted in the absence of any commercial or financial relationships that could be construed as a potential conflict of interest.
