# Supplementary material for: Bactericidal and Anti-biofilm Activity of the Retinoid Compound CD437 Against Enterococcus faecalis
Source: Front Microbiol. 2019 Oct 9;10:2301. doi: 10.3389/fmicb.2019.02301 (PMC6794434; doi:10.3389/fmicb.2019.02301)
Supplement: Supplementary file 1 [file Data_Sheet_1.pdf]

Supplementary Material

Table S1. Combined effect of CD437 with antibiotics on *P. aeruginosa* PAO1 biofilm formation.

Antibacterial agent	Class	MBIC (μg/ml)		Outcome
		Alone	Combination	
Gentamicin	Aminoglycoside	1	1	No interaction
Ceftriaxone sodium	Cephalosporin	128	128	No interaction
Levofloxacin	Quinolones	4	4	No interaction
Ciprofloxacin	Quinolones	1	1	No interaction

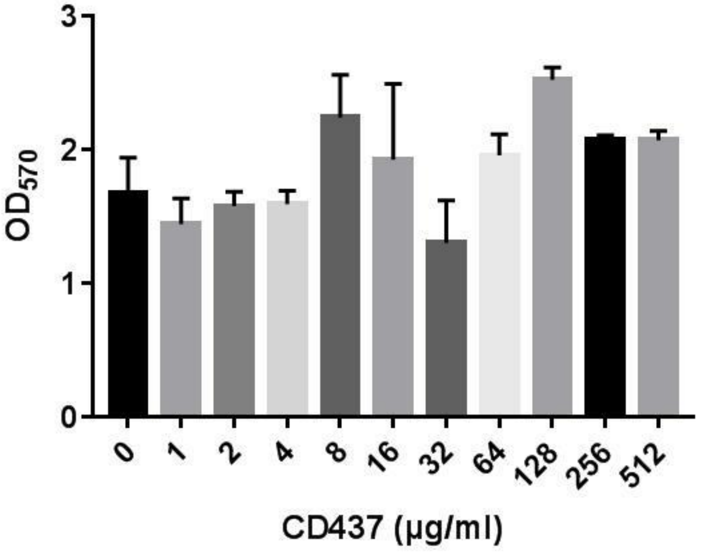

Figure S1. CD437 could not eradicate mature biofilm of *E. faecalis* ATCC 29212. Mature biofilm (24-h-old) was treated with CD437 for another 24 h and stained with crystal violet before quantification by measuring the absorbance at 570 nm. Data are presented as mean ± sd. The results are representative of three independent experiments.

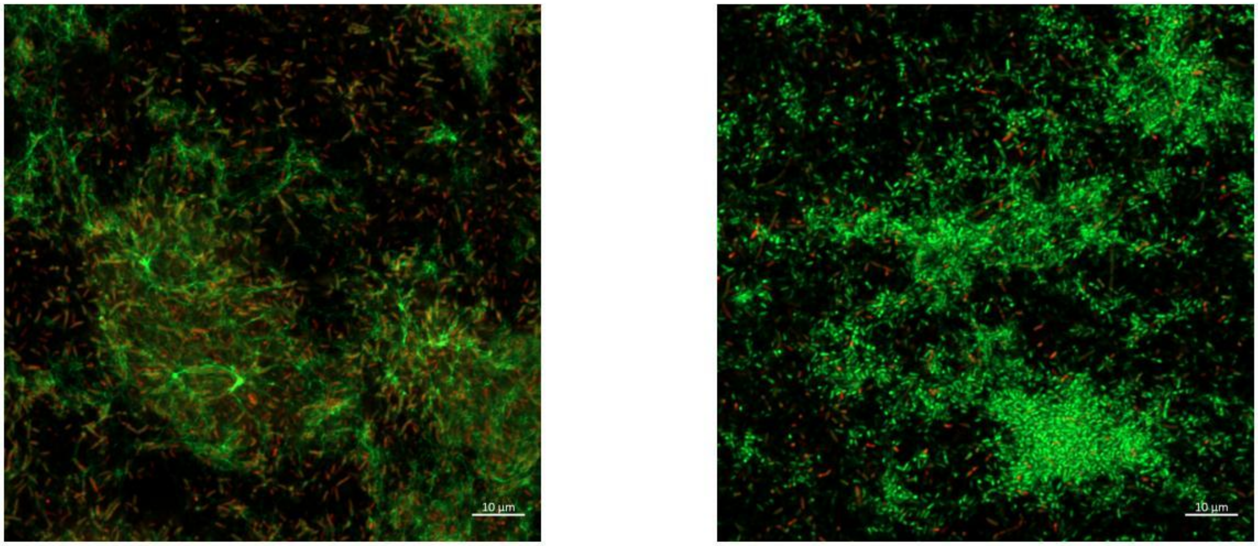

Control **CD437 (64µg/ml)**

Figure S2. CD437 changed the structure of PAO1 biofilm. Representative CLSM images of mature biofilms of *P. aeruginosa* PAO1 on glass slides treated with CD437 for 24 h. Biofilms were stained with SYTO9 (green, live bacteria) and PI (red, dead bacteria).

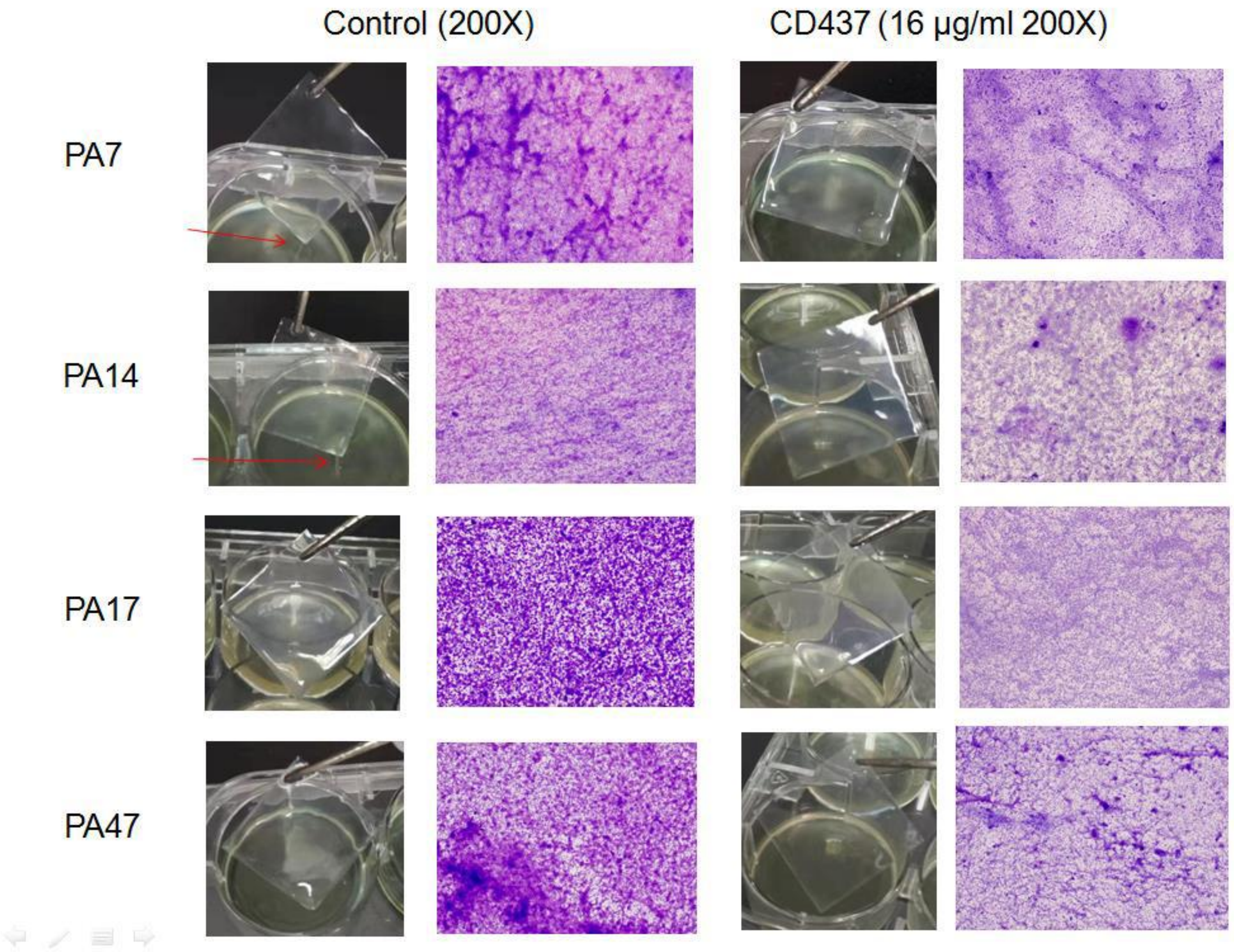

Figure S3. CD437 reduces the formation of *P. aeruginosa* clinical isolates biofilm matrix, but does not inhibit bacterial adhesion. Representative images of CD437 applied to the *P. aeruginosa* clinical isolates biofilms. Biofilms were stained with crystal violet .

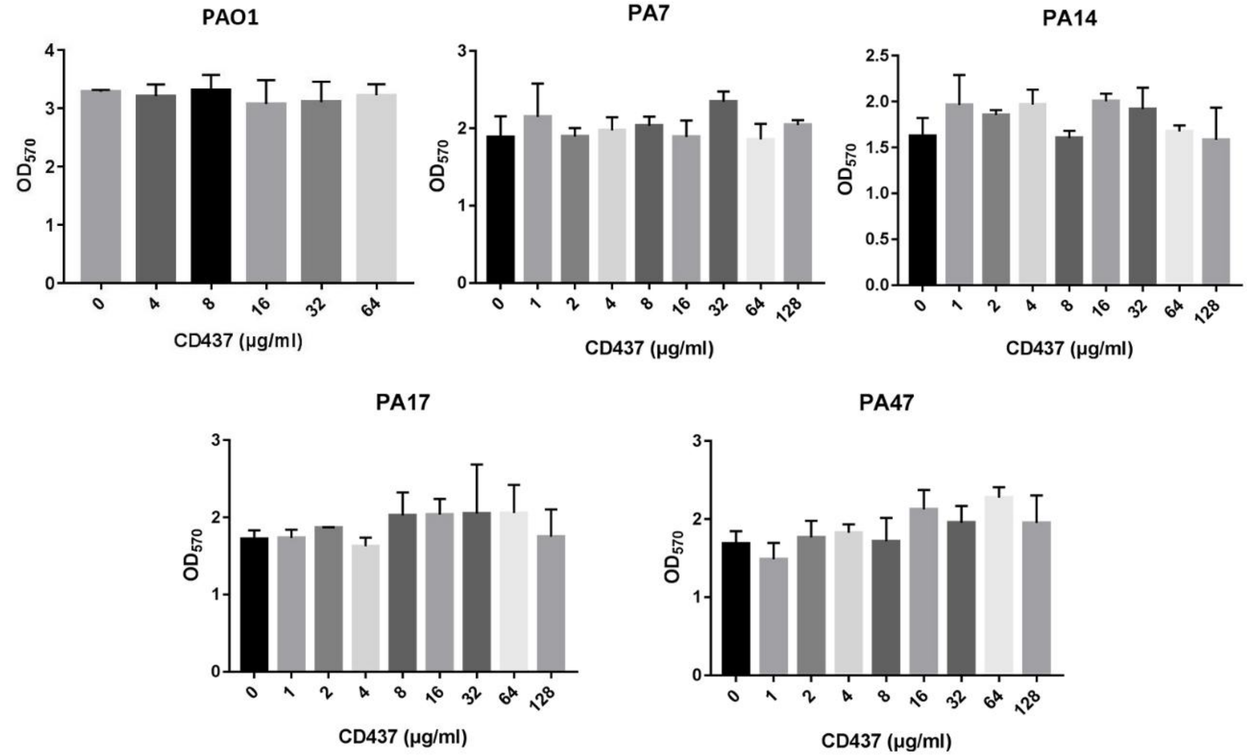

Figure S4. CD437 had no effect on *P. aeruginosa* biofilm biomass (crystal violet staining). Biofilms were cultured with or without CD437 for 24 h and stained with crystal violet before quantification by measuring the absorbance at 570 nm. Data are presented as mean ± sd. The results are representative of three independent experiments.
